# Podiatrists’ views and experiences of using real time clinical gait analysis in the assessment and treatment of posterior tibial tendon dysfunction

**DOI:** 10.1186/s13047-021-00482-8

**Published:** 2021-06-04

**Authors:** Paul Harradine, Lucy Gates, Cheryl Metcalf, Catherine Bowen

**Affiliations:** 1 The Podiatry Centre, 77 Chatsworth Ave, Hants PO6 2UH Portsmouth, UK; 2 University of Southampton, University Road , Hants SO17 1BJ Southampton, UK

## Abstract

**Background:**

Real time clinical gait analysis (RTCGA) is often incorporated as part of a general or lower limb musculoskeletal (MSK) adult patient assessment. However, it is not known if RTCGA is clinically effective as a useful outcome measure or aids in decision making. Whether there is a clinical worth in conducting RTCGA in adult MSK consultations remains controversial. The aim of this study was to provide unique insights into MSK podiatrists use and opinions of RTCGA, using Posterior Tibial Tendon Dysfunction (PTTD) as an exemplar adult condition.

**Methods:**

A qualitative approach was employed to explore MSK podiatrists’ views and experiences of RTCGA when assessing or treating patients with PTTD. Semi-structured interviews were conducted via Skype video calls which were transcribed using an orthographic transcription method. Thematic analysis was employed to identify key meanings and report patterns within the data.

**Results:**

Twenty nine MSK podiatrists who used RTCGA in the assessment and treatment of PTTD participated in the study. Five themes were identified as 1) RTCGA Method; 2) Working with RTCGA; 3) RTCGA uses; 4) What could aid RTCGA; 5) How RTCGA skills are acquired. This is the first known study to explore this topic of relevance to clinicians and researchers alike. Clinical observations were not only kinematic, but also included patient perceived experiences such as pain and orthotic comfort with normative kinematic reference values not perceived as important to that management goal. The most common barefoot RTCGA observations performed were the rearfoot to leg angle, medial bulge, forefoot abduction and arch integrity. However, a high amount of variation in many gait observations was noted between participants. Documentation methods also varied with a four-point scale system to grade motion and position most often employed and RTCGA was most often learnt through experience. The main barriers to performing RTCGA were clinical time and space restrictions.

**Conclusion:**

Findings from this study have provided a view of how podiatry MSK clinicians utilise RTCGA within their practice. MSK podiatrists use RTCGA as both an outcome measure and as an aid in decision making. This implies a perceived worth in conducting RTCGA, however further work is recommended that focusses on development of a national guideline to RTCGA to be adopted.

**Supplementary Information:**

The online version contains supplementary material available at 10.1186/s13047-021-00482-8.

## Background

Clinicians are often recommended to conduct gait analysis as part of a general or lower limb musculoskeletal (MSK) adult patient assessment [1–9]. The analysis of gait may be conducted with or without the use of computerised recording and analysis equipment to aid diagnosis, determine treatment goals and evaluate treatment outcomes [2, 6, 7, 10]. The frequency gait analysis is performed in routine clinical practice, with or without the use of equipment, is unknown but it has been suggested that most clinicians working in MSK clinics have limited access to computerised analysis or have time constraints limiting its use [2, 11, 12].

Real Time Clinical Gait Analysis (RTCGA) has previously been defined as assessment of gait conducted live in health professionals’ clinics, without the use of any recording, play back or computerised equipment [13]. Gait analysis conducted using computerised, recording or play-back equipment is termed Clinical Gait Analysis (CGA). A previous systematic review highlighted that the proportion of clinicians using RTCGA, how or why they use it, or in which situations, is currently unknown [13]. Furthermore, it is not known if RTCGA is clinically effective as a useful outcome measure or aids in decision making. There are also physiological limitations to RTCGA in that a human eye can only view and process limited images per second and so clinicians may miss critical events of the gait cycle, and the elements of perceived recollection causing issues in reliability and validity of observations. Whether there is still clinical value in conducting RTCGA in adult MSK clinics, therefore, remains controversial.

Although other professions will undertake RTCGA, this study focusses on MSK podiatrists based in the UK. MSK podiatrists, as a profession requiring the detailed knowledge of foot function, may perform RTCGA more frequently and therefore have greater experience and views to share. Bird and Payne, in Merriman’s Assessment of the Lower Limb [5], cite RTCGA as being one of the most widely used clinical assessments available. It has also been called a fundamental skill for podiatrists [8] and is a core subject in the UK Podiatric Medicine curriculum for undergraduate podiatry students [14].

Improving knowledge of MSK podiatrists’ views and experiences of RTCGA increases understanding of the reasons why, and methods by which, it is performed. An increase in knowledge may lead to further recommendations to aid the RTGA process. The aim of this study is to provide unique insights into some MSK podiatrists use and opinions of RTCGA, using Posterior Tibial Tendon Dysfunction (PTTD) as an exemplar common adult condition for which RTCGA is used.

## Methods

### Study design

This study has a descriptive phenomenological philosophical underpinning, using a qualitative methodology, featuring semi-structured in-depth one to one interviews to explore MSK podiatrists’ use and opinions of RTCGA in patients with PTTD.

This study was carried out in full accordance with the Declaration of Helsinki on ethical principles. Ethical committee approval was granted by the University of Southampton Ethics and Research Governance Committee (Reference: 55,599).

### Participants

MSK podiatrists were recruited for this study. MSK Podiatry is an area of expertise within the podiatry profession. However, there exists no professional definition of a MSK Podiatrist, and no specific qualification required to treat an adult MSK caseload in podiatry. Within this study MSK Podiatrists were defined following Vernon’s definition of expertise [15], as podiatrists with at least 5 years of MSK experience and consulting at least weekly with patients with lower limb MSK injuries. Sample size was determined following the recommendation by Braun and Clark [16]; a purposive sample of 30 MSK podiatrists was identified.

Participants were recruited using a specialist Facebook group (MSK:UK) following permission from the group administrator. A message briefly outlining the study was posted, inviting potential volunteers to contact the author via email (PH). A snowballing strategy through word-of-mouth was used to increase recruitment. All subsequent volunteers were sent an email introducing the research along with the participant information sheet, consent form and demographics form.

Recruitment strategy employed a purposive sampling framework to include Podiatrists with at least 5 years clinical experience in treating MSK patients, weekly consultations with adult patients with lower limb injury, access to a computer with Skype and an email address for correspondence. To prevent any financial or corporate bias in relation to gait analysis equipment Podiatrists with affiliations or involvement in any organisation or entity with any financial interest in CGA equipment were excluded.

The research team (PH, LG, CM, CB) agreed a priori to cease data collection if data saturation occurred before 30 interviews, such that saturation refers to a point were additional interview data fails to generate new information [17–20].

### Musculoskeletal condition of focus

PTTD was selected as a focus condition in this study. As a common adult MSK foot and ankle problem treated by podiatrists [21, 22], it was determined likely that MSK podiatrists would have a good awareness of the condition, diagnostic and treatment approaches. In addition, changes in kinematics (increased rearfoot eversion, forefoot abduction and arch lowering) are predictable and documented [22–26]. It is therefore a condition that MSK podiatrists will both be aware of, and most likely be using RTCGA, at all stages of the patient treatment pathway.

### Interviews

A semi-structured interview method was used to obtain rich and detailed data about individual experiences and perspectives of RTCGA [18]. Using this method responses are determined to a greater extent by the participant; issues important to them, but not specifically included on the interview guide, can be explored [27]. Interview questions were developed and approved by all authors. The interview guide is included as supplementary file 1.

Interviews were conducted by the author (PH) via Skype from the researcher’s and participants’ homes or places of work. This method of online face-to-face interviewing is more convenient for participants while still enabling observation of verbal and non-verbal cues [28]. It has been suggested that there is little difference in the data quality between online and face-to-face interviews, further supporting the use of online remote interviews [29, 30]. Transcription was completed by the author (PH) within 48 h of the interview, permitting ongoing assessment for saturation.

### Pilot study

The first 3 interviews were included in a pilot study; used for reflection and to ensure the technology and methodology was appropriate, and the information obtained was adequate.

No change to methodology was required, therefore these first 3 interviews were also included in the main data analysis.

### Data analysis

Data was transcribed by the author (PH) using orthographic transcription [31] and video files deleted. Completed transcripts were imported into a data analysis package (N-Vivo 2020).

Following transcription, an inductive approach to thematic analysis of the data was undertaken [16]. The codes, themes and interpretations of data were discussed and agreed within the research team. Respondent validity was then sought, with summarised data being reviewed by three participants to ensure results accurately reflected their intents and meanings [32].

## Results

Interviews were conducted between March and August, 2020. Interview duration ranged from 11 min to 52 s to 39 min and 9 s, with a mean duration of 19 min and 9 s. Saturation occurred at the 29th interview, when incoming data for the last 3 interviews produced no further new information [32]. No participants withdrew from the study.

Twenty-nine participants were therefore included in the study. All were based in the UK and practitioners in MSK podiatry with a range of characteristics described in detail in Table 1.

**Table 1:** Participant characteristics

Particpant	Year Qualified	Weekly MSK caseload (% of hours worked)	Weekly MSK caseload (hours)	Weekly MSK caseload (Number of patients seen)	Duration of MSK Speciality (Years)	Private practice (PP) / National Health Service (NHS) ratio	Additional Qualifications
1	2010	30	15	18	9	100 % PP	None
2	1994	100	45	70	25	80 % NHS 20 % PP	MSc Theory of Podiatric Surgery
3	2009	100	32	45	7	100 % NHS	MSc Podiatry
4	2000	80	28	40	18	100 % PP	None
5	2000	80	25	40	18	100 % PP	None
6	2009	100	38	50	11	95 % NHS 5 % PP	MSc Theory of Podiatric Surgery
7	2010	60	20	40	9	40 % NHS 60 % PP	None
8	2003	80	30	30	16	100 % PP	None
9	2008	100	37.5	32	10	50 % NHS 50 % PP	MSc MSK studies (lower limb)
10	1999	100	24	36	12	100 % NHS	MSc Clinical Biomechanics
11	1992	50	30	35	20	40 % NHS 60 % PP	PG Cert
12	1999	75	20	30	15	75 % NHS 25 % PP	MSc Theory of Podiatric Surgery
13	2014	25	8	10	5	100 % NHS	MSc
14	2005	70	25	20	10	20 % NHS 80 % PP	MSc
15	2001	75	30	22	17	100 % NHS	MSc Clinical Biomechanics
16	1994	90	45	40	25	100 % PP	MSc Podiatry
17	1992	40	12	25	28	100 % PP	None
18	1988	10	4	5	32	100 % NHS	None
19	1987	25	10	10	30	100 % PP	None
20	1996	20	6	4	20	100 % PP	None
21	2009	30	6	7	11	100 % PP	PG Dip
22	1990	100	35	60	30	100 % NHS	MSc Clinical Biomechanics
23	2007	80	22	32	13	100 % PP	None
24	2002	100	19	28	15	100 % NHS	None
25	1991	50	17.5	16	30	100 % PP	PhD
26	2003	15	3	3	13	100 % PP	None
27	2003	80	28	28	17	10 % NHS 90 % PP	None
28	2003	100	25	30	15	100 % PP	MSc Sports Podiatry
29	1995	98	35	80	25	100 % PP	PG Dip Biomechanics
Average	2000	68	23	31	17	62 %PP 38 %NHS	NA

 The three participants contacted for respondent validity confirmed the study results accurately reflected their intents and meanings.

Five key themes were identified using thematic analysis: (1) RTCGA Method; (2) Working with RTCGA; (3) RTCGA uses; (4) What could aid RTCGA; (5) How RTCGA skills are acquired. An abridged summary, with excerpts of data drawn from the transcripts, is presented below. Non-verbal utterances (e.g. er, erm), repeated words and thinking pauses have been removed to improve the reading process. A Word Cloud, to demonstrate word frequency, is presented as supplementary file 2.

### Theme 1: RTCGA Method

This theme explains RTCGA methods and procedures. This includes not only the physical observation itself, but also how the findings are documented.

All participants reported performing RTCGA at some time, even if they also had access to equipment or technology to perform CGA, however the processes employed varied between participants. Commonly the RTCGA process begins when the patient walks into the clinic, allowing an observation thought to be more valid as a relaxed unobserved gait pattern.

*“So when I do gait analysis to start with I always collect the patient from the waiting room so I look at how they get up, how they walk back to the clinic because that is a little bit of a walk and they get in front of me and that’s usually quite good because although they’ve got their shoes on they’re not self conscious”* (Int 15).

When the process of RTCGA is performed and the patient is aware of the observation, participants reported that they will assess their patient walk in the best environment they have available to them. This appears rather opportunistic, with clinics, doorways and corridors being used, depending on room and the confidentiality of the setting. The procedure, in terms of how long patients walk, podiatrist position, method of observations and if they walk barefoot or shod first, varied between participants.

*“And they will walk to and fro from me either in a hallway, corridor, room, waiting area. Wherever is free hopefully not observed by other people”* (Int 2).

The timing of when RTGCA was performed during a patient assessment showed no common trend across the participants. Dynamic observations occurred before or after static analysis. In addition, there emerged no common order to which specific observations were conducted.

*“So, I just get a patient to get up and just start walking. I use a short space. Probably be about three meters that’s about the length of the corridor and I’ll just say to the patients nice and relaxed and can you just walk back and forward at your own constant pace and that’s it really. I tend to try to be kind of systematic in what I’m doing. I’ll always look at the foot straight away and then I’ll tend to work my way up and have a look at the knee position if I can get their trouser legs above their knee”* (Int 14).

*“I generally will ask the patient to leave their shoes on when they arrive and ask them to walk up and down. I’ll note what I’m seeing. I’ll then ask the patient to sit down and go through the problem in a bit more depth and then I’ll go unshod”* (Int 17).

CGA, utilising technology and equipment, was used occasionally. The most frequent equipment utilised being 2D video analysed using computer software. Participants who used this method reported that it was used after RTCGA had been conducted.

*“I generally watch them walking usually across a room first. I feel you get more from that than from anything else. If there’s something in particular I want to get in more detail, I would then video them. Occasionally you can’t video them because their mobility isn’t good enough. And if I wanted to show them something I would also video them”* (Int 8).

Participants varied in their account of their method or structure employed when documenting RTCGA findings. Grading systems were most commonly used, the most prevalent being to grade motion or positions via a four-point scale of none, small, medium or large.

*“Yeah I’d generally go small, medium, large. Generally on grading scales I use a three point or a five point grading. I don’t see the point of getting too much finer than that”* (Int 23).

*“Large, medium or small or none, yeah*” (Int 15).

There is a high amount of variability in the RTCGA observations. For barefoot RTCGA there were a total of 132 different observations noted across the participants, 82 of which were individual (observed by only one of the participants interviewed). Only four observations occurred commonly (rearfoot to leg, medial bulge, forefoot abduction and the arch). Shod assessment, occurring either with or without orthotics at either assessment or review, demonstrated less total variation of observations across participants. 62 shod observations were stated, less than half of those used for barefoot RTCGA. Again, there is a high amount of observations individuality, with 43 of 63 observations noted only to occur once across participants. Only two observations were noted commonly: the medial bulge (most frequent) and the rearfoot to leg angle (less frequent). These two observations were also noted in the barefoot RTCGA observations, but when barefoot, the rearfoot to leg presented as a more common trend than the medial bulge observation.

*“No. You clearly aren’t. You’re looking at the shoe. But then what I’m tending to look for is that kind of splay of the shoe to the medial side. The kind of bulging out on the medial side.”* (Int 20).

### Theme 2: Working with RTCGA

This theme explores how participants work with RTCGA for PTTD in the environment of a lack of formal instruction [13].

Even though all participants used RTCGA in the assessment of patients with PTTD, there was a common acceptance that quantitative objective measurements were not possible and that the process was a subjective one.

*“It’s more you just write what you see there’s not really a structure to it you get when you follow a system it’s really kind of what you see. It’s very subjective”* (Int 13).

Participants valued RTCGA in clinical decision making. When a RTCGA outcome was discussed, such as a decrease in rearfoot eversion or reduction in the medial bulge, participants would use this result to guide further treatment. For example, if a patient’s symptoms had not improved but their RTCGA demonstrated a positive change, the participant stated that they would often refer on for further imaging. If asked if they trust their gait analysis, the answer was yes.

*“Yeah I probably am”* (Int 2).

*“That is what I do currently yes”* (Int 3).

The majority of participants highlighted a lack of established normative kinematic data to use in relation to RTCGA. Instead, observations were performed in relation to the presenting symptom.

*“I don’t know what normal or abnormal is”* (Int 4).

*“I certainly wouldn’t be using a zero degree you know the calc being completely vertical to the tibial. I don’t tend to use a reference point. The idea that for me is just to try to reduce that stress, reduce that calcaneal eversion. More obviously you’re reducing force more. But I don’t use a reference point to try to get it up to no”* (Int 14).

Participants did consider footwear a limitation to conducting RTCGA. Observing shod walking was seen as a restricted observation, and indication of in-shoe foot function or a direct observation of footwear only.

*“You might see certain markers or indicators. You might see sort of the medial heel counter or the upper you may see some movement there. But definitely you know barefoot you will see them, you’ll get a much clearer view of that arch flattening”* (Int 22).

If using observation of the shoe to infer in-shoe foot motion, the limitations of this approach were often acknowledged.

*“we don’t necessarily know what the foot’s doing inside the shoe but if I don’t see the shoe evert I assume that the foot’s not everting anymore. I know now that isn’t super accurate but then again it’s best can we do clinically”* (Int 28).

### Theme 3: RTCGA uses

This theme describes different uses of RTCGA through stages of the patient treatment pathway.

Participants reported that RTCGA was most commonly used at assessment and foot orthoses fitting, and much less so at review. Participants used RTCGA to assess kinematics, which link to the symptom, but also to reproduce gait-related pain in the clinic to aid in the assessment and diagnosis of the injury.

*“So, wanting to obviously look at the hip position, the knee positioning the foot positions seeing whether there was any abnormalities which you may be able to pick up on. Any limping, any pain.”* (Int 13).

Commonly RTCGA was used to assess kinematic outcomes after the provision of orthotics, both positive or negative, and to check footwear suitability.

*“Again, I’m probably looking at the calcaneus seeing if that is, if the valgus is reduced, again if the talonavicular joint is less visible. Probably the main things with a shoe on I’d be looking at would be from behind sort of rearfoot to leg angle really”* (Int 20).

*“Yea, that would be barefoot really and then whatever I do and put in their footwear it’s more or less just to see, is the shoes helping things. Is the particular footwear maybe not suitable and then you could advise the patient of that? I’m also seeing is it correctable by putting the patient in footwear, does it make a difference to the foot position that you can see as they walk along?”* (Int 7).

*“just to make sure we aren’t causing any other complications”* (Int 27).

RTCGA was occasionally stated as being invalid to assess kinematic changes with foot orthoses.

*“I don’t think I’ll see as much with the shoe on”* (Int 11).

*“No. Because kinematics is a blunt instrument”* (Int 25).

Following the fitting of orthotics, RTCGA was often used to check orthotic comfort. This trend was stronger than using RTCGA to assess for kinematic changes.

*“To be honest as soon as I put the insoles in, I’m more looking for the comfort aspect. I don’t really look to see if anything in gait is changed”* (Int 14).

CGA was occasionally used for kinematic comparison at review appointments and to encourage patient engagement in treatment plans.

*“They also see the technology analysis so they get to see their footscan images, they get to see the video gait analysis, you explain what’s going on and you actually have a visual to back that up rather than just saying oh as I watched you walk there I can see X, Y and Z. So perhaps the patient involves themselves a wee bit more in treatment and gains more confidence in you as a practitioner with those visual aids there”* (Int7).

### Theme 4: What could aid RTCGA?

This theme reports views on changes that may be beneficial to MSK podiatrists using RTCGA. It brings together possible clinical factors, which limit or restrict RTCGA.

When participants were asked what they believed the challenges and difficulties were regarding RTCGA, some suggested that a more standardised approach would be helpful.

*“I suppose it would be quite nice to have a set, sort of not rules as such, but something to follow so you know, perhaps in different parts of gait, things to look out for. Kind of like the FPI [Foot Posture Six Index] I suppose”* (Int 1).

*“I would love to have one, that you know, we could all say every single podiatrist in the UK uses, a standardised one, but I’m not aware one exists. But it would be great if it did exist”* (Int 7).

Occasionally specific areas of improvement such as measurement and documentation were suggested. In addition, while discussing the possible benefits of new approaches specific concerns were noted. These included any suggested changes not taking more time than currently available, fitting in with their current method and that it had undergone testing for validity and reliability.

*“I’d use it as guidance. And if it if it worked and it fitted in and it was a good way to document something to get decent values that were easily understood and reliable between different podiatrists.”* (Int 19).

A lack of clinical space and time were noted as barriers to RTCGA.

*“Well really just that you know when you’re watching them walk down a corridor you can only see them from the front and behind so you’re not getting a true look at the talonavicular bulging and the reduction of the arch profile you can only observe that when they’re right in front of me so that’s something that, as they closer to me, I’ll angle myself and have a little sneaky peak. Whereas when you’re looking from the front and back, all you’re seeing is you’re not getting a sagittal plane really because you’re unable to see from the side, so I’d say that’s my biggest limitation. You could always look at it as a time issue as well.”* (Int 14).

Use of gait assessment equipment or technology was suggested by the majority of participants. Both positive and negative opinions were present. It was not seen as a necessity.

*“I wasn’t convinced by a lot of it. That’s my personal opinion and probably I’m shooting myself in the foot here but I think you can get very bogged down in things, machines telling you what should be wrong with that patient”* (Int 10).

*“Yes, well I guess in the NHS its very much the visual thing because we don’t really have the facilities here to record the information and retain the information from the video analysis, but in my private practice I would use more the video analysis and the FScan read outs and information because at least that can be saved and documented and then you can come back to it after treatment”* (Int 7).

### Theme 5: How RTCGA skills are acquired

This theme relates to how participants have obtained their knowledge of the methods and reasons for performing RTCGA.

Most participants indicated that they acquired RTCGA skills via experience. Some also learnt from colleagues, post and undergraduate education, journals and books. However, the occasionally stated the use of literature and courses was expressed only in conjunction with experience.

*“It’s down to experience and it’s down to knowing what I feel is normal based on what I’ve seen over the years and what I’ve assessed”* (Int 18).

*“I think over thirty years having to listen to many lectures and read many papers and seen many patients I’ve collated lots of sort of little pearls of wisdom from many different practitioners and I use that to really give me my full understanding of the injured area, patients function, and where the patient wants to be and how I can get them there.”* (Int 17).

## Discussion

The results of this study provide unique insight into these MSK podiatrists experience and opinions of RTCGA (focusing on PTTD as the exemplar). Five themes emerged as (1) RTCGA Method; (2) Working with RTCGA; (3) RTCGA uses; (4) What could aid RTCGA; (5) How RTCGA skills are acquired.

Participants in this study used RTCGA in the assessment and treatment of PTTD, in accordance with recommendations that gait analysis forms part of a general or lower limb MSK adult patient assessment [1–9]. It was evident that their observations of gait included kinematic scrutiny combined with patient-perceived experiences such as pain and orthotic comfort. In addition, the observation sequence was variable and normative reference values for gait were found to be generally unimportant.

The most common barefoot RTCGA observations performed were the rearfoot to leg angle, medial bulge, forefoot abduction and arch integrity. These kinematic observations are markers of pronation [33], changes of which are acknowledged in PTTD [22–26]. Only two common RTCGA observations emerged relating to the shod context: the rearfoot to leg angle and medial bulge. The main reason for this was explained by participants as the challenge in observing the foot when the shoe obstructs visualisation of the foot movements. These two observations within the shod context were however still used as markers of pronation, where assessing shoe kinematics was seen as a proxy marker of foot function. Conversely, findings suggest MSK podiatrists are aware of the limitation in validity of inferring in-shoe foot motion from footwear observation [34].

The two common shod observations (the rearfoot to leg angle and medial bulge) allowed participants to theoretically assess the immediate kinematic outcome of management strategies to modify pronation, such as at the fitting of foot orthoses. RTCGA was not used to assess kinematic outcomes over longer time periods, such as between appointments. Moving patients away from painful gait patterns was expressed as the primary rehabilitation objective by participants in this study. These observations and methods are in agreement with recommendations from other authors to reduce pronation as an aim of treatment for PTTD [21, 22, 35]. The observations from participants in this study also highlighted a lack of clinically feasible and reliable normative kinematic data to use in relation to RTCGA and support the reasoning towards a focus on the presenting clinical symptoms rather than modification of risk factors for preventative approaches.

Although common kinematic observations emerged, a high amount of variation was also noted between participants in this study. A variety of anatomical landmarks, motions and terminology were described. This was not expressed as a problem and participants did not appear to be aware that their observations were often individualised. Documentation of RTCGA findings was also diverse, the most common method being to grade motion or positions via a four-point scale of none, small, medium or large. The high number of variations in observations and documentation may be a result of RTCGA lacking structured and validated guidelines [13]. Indeed, some participants suggested that a standardised approach would be helpful, indicating that future work should focus on development of national guidelines through expert consensus.

CGA - utilising technology and equipment - was used occasionally by participants in this study. The most frequent equipment reported was 2D video analysed through computer software. CGA equipment was not deemed as being essential to assess the gait of patients with PTTD, but the benefits for its use were expressed by some and countered by others. The negative opinions presented in this study are contrary to the positive emphasis on CGA found in literature, where it has been suggested that CGA is more efficacious, valid and reliable than RTCGA [2, 36, 37]. The reason for the participant’s differing opinions on the use of CGA were not specifically explored as part of this study. However, it could be in part explained by appropriateness of the clinical setting and a lack of time being cited as challenges and barriers to RTCGA. Likewise, there was a common acceptance that quantitative objective measurements were not possible and that the process was a subjective one. This is not surprising given the inadequacies of nationally agreed guidance for minimal RTCGA space and time requirements [13].

The final theme related to how RTCGA skills were acquired primarily through experience and occasionally via observing colleagues, courses and review of literature. Learning through experience, or ‘experiential learning’, is a well-established theory relating to teaching and skill acquisition [38]. Although it is acknowledged that practical skills can be taught with learner participation, the delivery of good experiential learning has become complex, possibly even ‘super complex’ [39]. Itin [40] state the educators main role in experiential learning include selecting suitable experiences for the learner whilst posing problems, setting boundaries, supporting, insuring physical and emotional safety, guiding reflection and providing any necessary information. This may be difficult to perform in a large group or classroom environment. Instead RTCGA skill acquisition may be best suited to a small group or even a mentoring system. However, in the context of learning RTCGA the participants in this study often described their experience as the unique method by which they have acquired the skill or knowledge to perform RTCGA. They are not being purposely ‘taught’ a method from best practice nor is it evidence-based. A potential explanation is the current lack of RTCGA research and literature [13], or that simply experiential learning is the best way to acquire skill in this area.

### Potential Strengths and Limitations

This study is the first of its kind to investigate MSK podiatrists use and opinions of RTCGA.

That said, a number of limitations should be considered when interpreting the findings. Firstly, this study examined the perceptions of MSK podiatrists currently practising in the UK. Results may not, therefore, be representative of MSK podiatrists from other countries or to other professions who perform RTCGA. In addition, other than being in the UK, geographical details of the participants are not known. It is possible different areas in the UK may have other uses and opinions on RTCGA not represented in this study.

Secondly, a purposive sample was used, where first responders were selected. It is not known if the range of participant characteristics (Table 1) is a fair representation of MSK podiatrists working in the UK. However, this method allowed for an efficient gathering of primary data regarding RTCGA.

Thirdly, the 1:1 interview process may lead to a lack of research breadth due to smaller sample sizes (when compared to, for example, a large-scale survey) [16]. These limitations reduce confidence that results can be generalisable as representative of all UK MSK podiatrists. Although saturation was reached within the study sample, demonstrating no new data may have been forthcoming with an increased sample size, generalisability was not the intention of this study. Rather it was to achieve in-depth insight from the purposively sampled participants. The 1:1 interview process allowed the collection of rich and detailed data with flexibility to pursue different areas or subjects as they arose. This is valuable with inductive research in circumstances such as this, where there is a lack of established literature and knowledge [16].

Fourthly, information regarding the success of the different recruitment strategies to obtain participants was not collected. Volunteers were obtained via a specialist Facebook group (MSK:UK) or through a snowballing strategy via word-of-mouth. Awareness of the most efficient recruitment strategy may have been helpful for researchers conducting comparable research. Finally, PTTD was used as focus condition in this investigation. This allows for confidence in relevance of findings to this condition but restricts the universal application of results to other MSK lower limb injuries. Relevance to general MSK podiatry caseloads is therefore limited. However, this study could be used as an exemplar model from which views and opinions on RTCGA and other lower limb conditions can the investigated.

### Further recommendations

MSK podiatrists use RTCGA when treating adult PTTD as both an outcome measure and as an aid in decision making. This implies a perceived worth in conduction of RTCGA. Further research incorporating wider sampling and investigating other MSK lower limb conditions appears justified.

An improvement in clinical space and time is advocated to facilitate RTCGA. However, the actual amount of time and space required for RTCGA has not been established. It is recommended that minimum requirements are established in relation to the clinical environment and appointment durations best suited for RTCGA. Further suggestions of applying such minimum requirements to aid in RTCGA can then be advised.

The availability of a standardised approach would be seen as a positive aid to RTCGA and PTTD. It is recommended that the development of a standardised approach, such as the creation of national guidelines through expert consensus and stakeholder involvement, is undertaken. Findings from this study can be used to aid any such future development.

The findings of this study have provided unique insight into how these UK based MSK podiatrists utilise RTCGA in practice. A more comprehensive representation of the use of RTCGA maybe achieved by further work such as an international survey.

## Conclusions

Findings from this study have provided a comprehensive view of how podiatry MSK clinicians utilise RTCGA within their practice. RTCGA is used regularly by MSK podiatrists as an outcome measure and to aid decision making when assessing and treating adult PTTD. This implies a perceived worth in conducting RTCGA from a Podiatric viewpoint. Observations were not merely kinematic, but also included patient perceived experiences, such as pain and orthotic comfort. Common kinematic observations emerged for both the barefoot and shod context. The main difficulty in performing RTCGA is restriction on clinical time and space, while a more systematic approach to RTCGA would be seen as helpful. RTCGA is a skill acquired through experience. Further work is recommended that focusses on development of national RTCGA guidelines.

## Supplementary Information

**Additional file 1:**

**Additional file 2:**

## Data Availability

The anonymised data that support the findings of this study are available from the corresponding author upon reasonable request.

## Abbreviations

CGA: Clinical Gait Analysis
MSK: Musculoskeletal
PTTD: Posterior Tibial Tendon Dysfunction
RTCGA: Real Time Clinical Gait Analysis
