# Supplementary material for: Podiatrists’ views and experiences of using real time clinical gait analysis in the assessment and treatment of posterior tibial tendon dysfunction
Source: J Foot Ankle Res. 2021 Jun 4;14:42. doi: 10.1186/s13047-021-00482-8 (PMC8176680; doi:10.1186/s13047-021-00482-8)
Supplement: Supplementary file 1 — Additional file 1: [file 13047_2021_482_MOESM1_ESM.docx]

Interview Question Guide

**Q1**: When assessing and treating adults with posterior tibial tendon dysfunction, do you watch and assess their walking?

**Q2**: If no, why not?

**Q3**: If yes, what are you reasons and aims of doing this?

**Q4**: Are there any challenges or difficulties with assessing their walking?
