# Supplementary figures and images for: Podiatrists’ views and experiences of using real time clinical gait analysis in the assessment and treatment of posterior tibial tendon dysfunction
Source: J Foot Ankle Res. 2021 Jun 4;14:42. doi: 10.1186/s13047-021-00482-8 (PMC8176680; doi:10.1186/s13047-021-00482-8)

Supplementary file 2. A word cloud to dmeonstrate word frequency


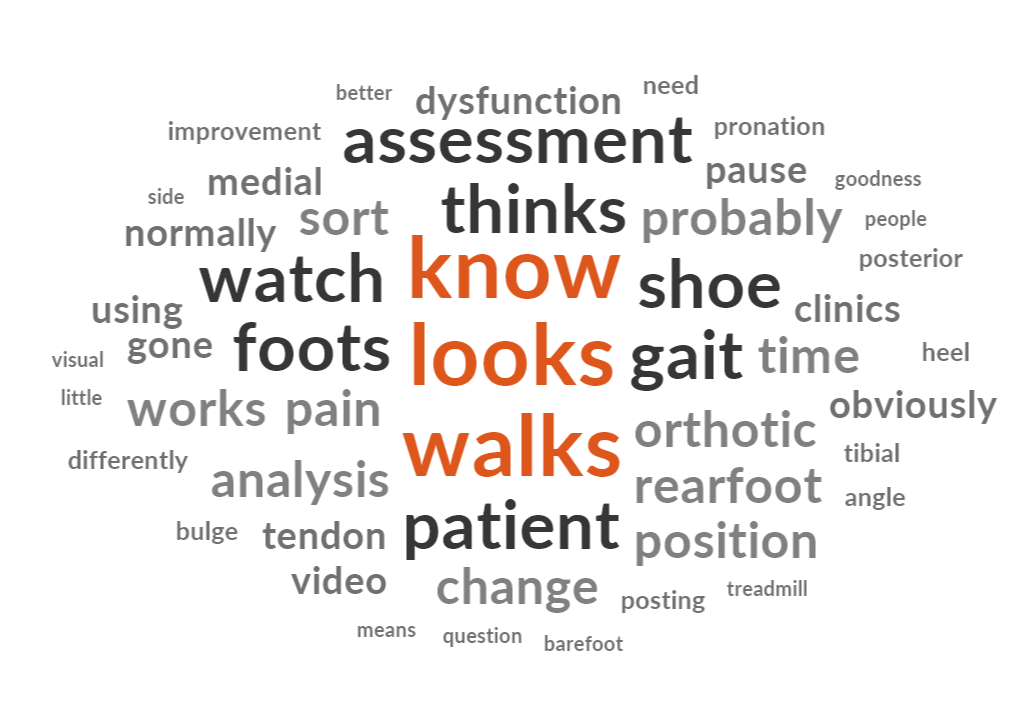

Supplement: Supplementary file 2 — Additional file 2: [file 13047_2021_482_MOESM2_ESM.docx]
